# Supplementary material for: Piezo1 Channels Contribute to the Regulation of Human Atrial Fibroblast Mechanical Properties and Matrix Stiffness Sensing
Source: Cells. 2021 Mar 16;10(3):663. doi: 10.3390/cells10030663 (PMC8002259; doi:10.3390/cells10030663)
Supplement: Supplementary file 1 [file cells-10-00663-s001.pdf]

cells

MDPI

Supplementary Material

Piezo1 Channels Contribute to the Regulation of Human Atrial Fibroblast Mechanical Properties and Matrix Stiffness Sensing

Ramona Emig [1,2,3,†], Wiebke Knodt [1,†], Mario J. Krussig [1], Callum M. Zgierski-Johnston [1], Oliver Gorka [4], Olaf Groß [4,5,6], Peter Kohl [1,2], Ursula Ravens [1] and Rémi Peyronnet [1,*]

1 Institute for Experimental Cardiovascular Medicine, University Heart Center Freiburg · Bad Krozingen, and Faculty of Medicine, University of Freiburg, 79110 Freiburg, Germany; ramona.emig@universitaets-herzzentrum.de (R.E.); wiebke.knodt@gmail.com (W.K.); mkrussi@g.clemson.edu (M.J.K.); callum.michael.johnston@universitaets-herzzentrum.de (C.M.Z.-J.); peter.kohl@universitaets-herzzentrum.de (P.K.); ursula.ravens@tu-dresden.de (U.R.)
2 CIBSS Centre for Integrative Biological Signalling Studies, University of Freiburg, 79104 Freiburg, Germany
3 Faculty of Biology, University of Freiburg, 79104 Freiburg, Germany
4 Institute of Neuropathology, Medical Center-University of Freiburg, Faculty of Medicine, University of Freiburg, 79106 Freiburg, Germany; oliver.gorka@uniklinik-freiburg.de (O.G.); olaf.gross@uniklinik-freiburg.de (O.G.)
5 Signalling Research Centres BIOSS and CIBSS, University of Freiburg, 79104 Freiburg, Germany
6 Center for Basics in NeuroModulation (NeuroModulBasics), Faculty of Medicine, University of Freiburg, 79104 Freiburg, Germany
* Correspondence: remi.peyronnet@universitaets-herzzentrum.de
† These authors contributed equally.

Citation: Emig, R. Piezo1 Channels Contribute to the Regulation of Human Atrial Fibroblast Mechanical Properties and Matrix Stiffness Sensing. *Cells* **2021**, *10*, 663. https://doi.org/10.3390/cells10030663

Academic Editor: Cord Brakebusch, Kate Møller Herum

Received: 25 January 2021
Accepted: 12 March 2021
Published: 16 March 2021

Publisher's Note: MDPI stays neutral with regard to jurisdictional claims in published maps and institutional affiliations.

Copyright: © 2021 by the authors. Licensee MDPI, Basel, Switzerland. This article is an open access article distributed under the terms and conditions of the Creative Commons Attribution (CC BY) license (http://creativecommons.org/licenses/by/4.0/).

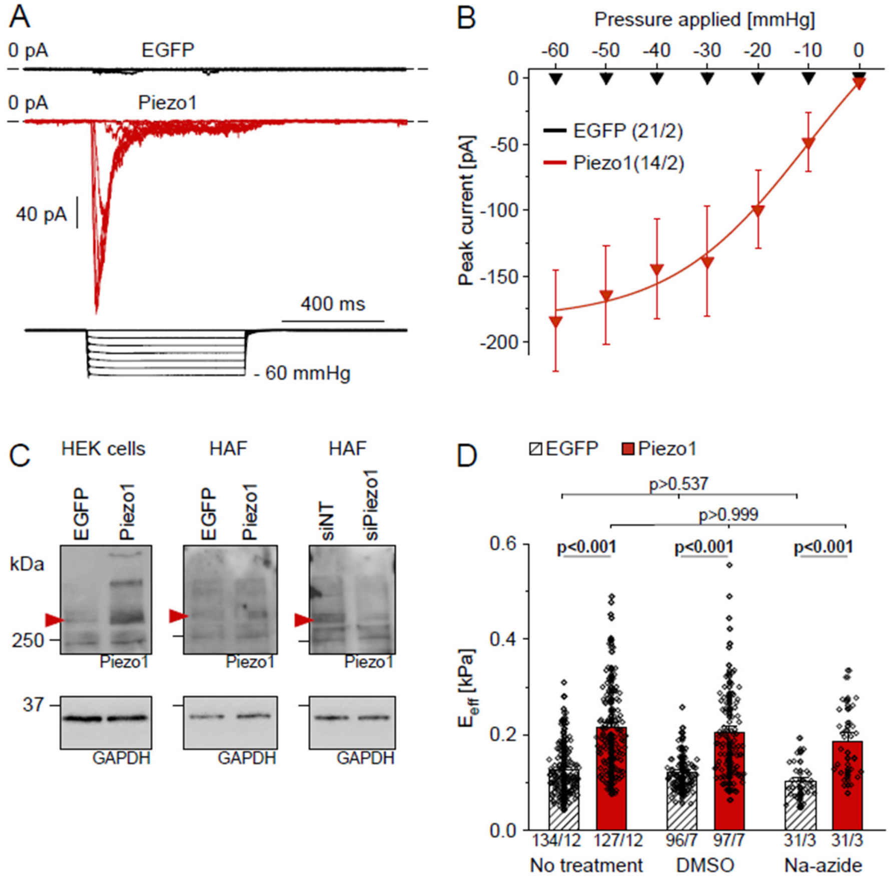

Figure S1. Overexpression of Piezo1 results in higher stretch-induced current, protein level and cell stiffness. (**A**) stretch-activated channel activity was recorded with the patch-clamp technique

in cell-attached mode at −80 mV 3 days post-transfection. Channel activity was elicited by pulses of negative pressure (bottom trace) applied through the patch pipette to stretch the membrane of HEK cells overexpressing EGFP (top trace) or Piezo1 (middle trace). (**B**) corresponding peak currents for all pipette pressures tested (from 0 to −60 mmHg) with EGFP or Piezo1. (**C**) whole cell lysates of HEK cells and HAF were prepared 3 days post-transfection of EGFP, Piezo1, non-targeting siRNA (siNT) or Piezo1-targeting siRNA (siPiezo1), separated by polyacrylamide gel electrophoresis, and immunoblotted for Piezo1 and glyceraldehyde-3-phosphate dehydrogenase (GAPDH). Red arrow heads indicate the expected position of monomeric Piezo1 slightly above 250 kDa. (**D**) Comparison of cell stiffness from all control groups that were either not treated at all, or treated with the solvents of the compounds used in this study (Table 1). DMSO was used at 0.1% (*v*/*v*) and Na-azide at 0.09% (*v*/*v*). n/N = number of cells/ number of experiments.

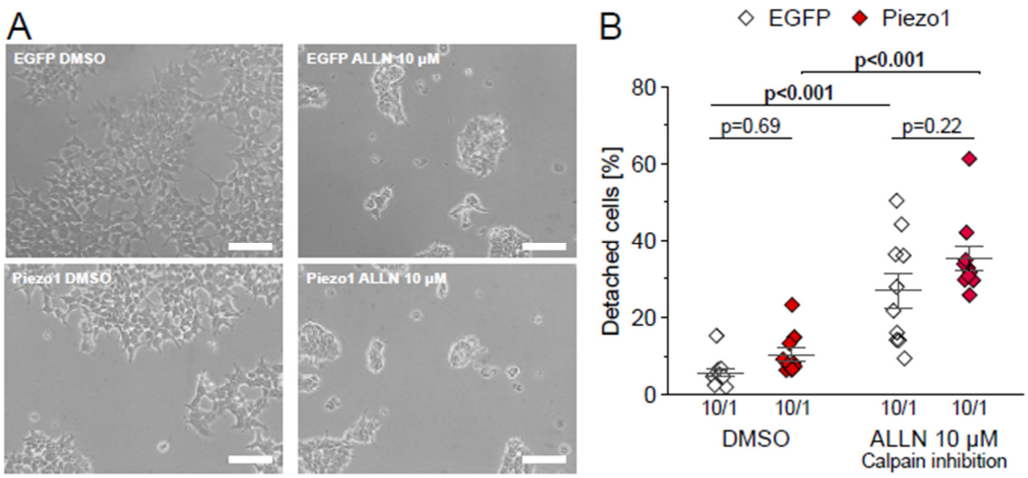

Figure S2. Calpain-inhibition leads to changes in cell morphology and detachment in HEK cells. (**A**) representative images of cells treated with DMSO or 10 µM ALLN or for 48 h. (**B**) quantification of cell detachment in response to treatment with DMSO or 10 µM ALLN. n/N = number of images/ number of experiments.

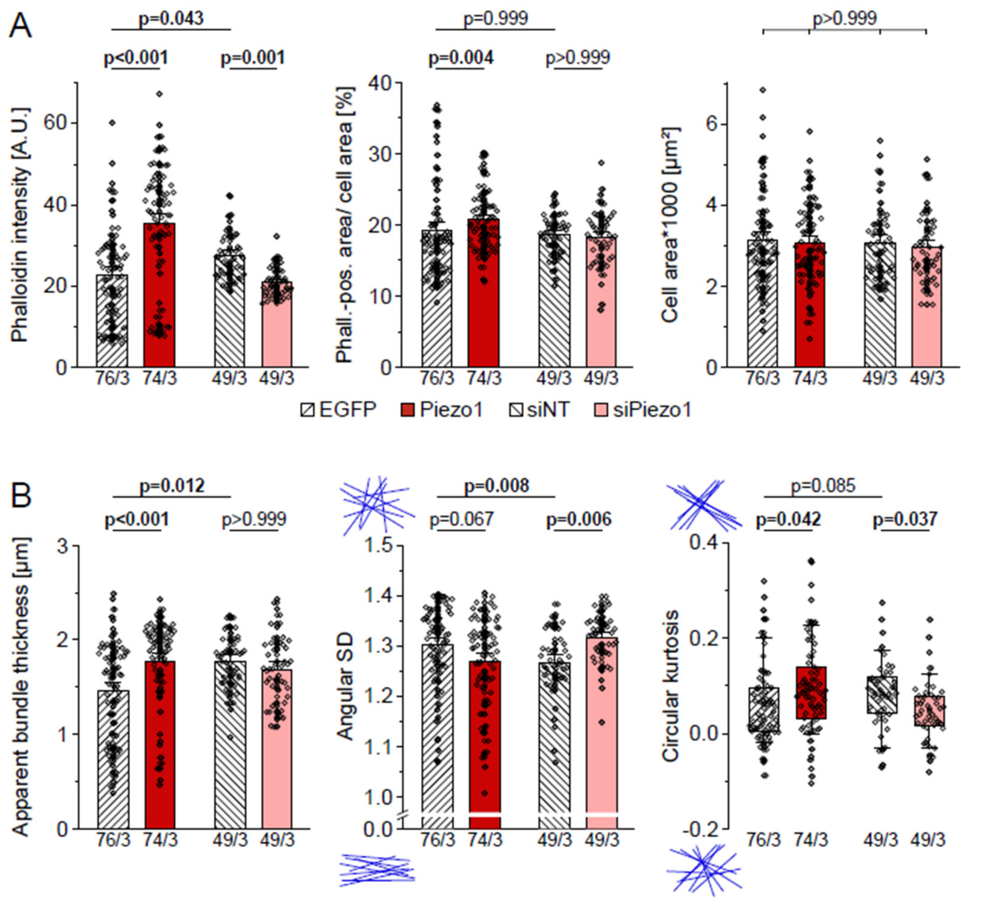

Figure S3. Piezo1-dependent alterations of the stiffness of HAF are the result of differential organization of the actin cytoskeleton. (**A**) average phalloidin intensity, phalloidin-positive area per cell area, and cell area. (**B**) apparent thickness, angular standard deviation (SD) and circular kurtosis of actin bundles. n/N = number of cells/ number of experiments.
